# Identification of tumor-associated antigens with multi-cancer therapeutic potential

**DOI:** 10.3389/fimmu.2026.1839980

**Published:** 2026-06-05

**Authors:** Daniel P. Wickland, Erik Jessen, Asha Nair, Brian Necela, Kimberly Lauer, Kiran K. Mangalaparthi, Rex Devasahayam Arokia Balaya, Akhilesh Pandey, Aaron S. Mansfield, Keith L. Knutson, Yan W. Asmann

**Affiliations:** 1 Department of Quantitative Health Sciences, Mayo Clinic, Jacksonville, FL, United States; 2 Department of Quantitative Health Sciences, Mayo Clinic, Rochester, MN, United States; 3 Department of Immunology, Mayo Clinic, Jacksonville, FL, United States; 4 Department of Laboratory Medicine and Pathology, Mayo Clinic, Rochester, MN, United States; 5 Center for Individualized Medicine, Mayo Clinic, Rochester, MN, United States; 6 Manipal Academy of Higher Education, Manipal, Karnataka, India; 7 Department of Medical Oncology, Mayo Clinic, Rochester, MN, United States

**Keywords:** antigen, cancer, HLA, immunopeptidome, immunotherapy

## Abstract

Tumor-associated antigens (TAAs) are non-mutated antigenic peptides expressed in cancer tissue at abnormally high levels but in normal tissue either at negligible levels, during only particular developmental stages, or in a tissue-restricted manner. The shared nature of TAAs across patients makes them attractive targets for off-the-shelf immunotherapies. However, no studies have comprehensively surveyed the TAA potential of all possible wild-type peptides originating from cancer-associated genes. In this study of 16 cancer tissue types and 39 normal tissue types, we analyzed the expression profiles of protein-coding genes to identify those with aberrantly high RNA levels across multiple solid tumor types and low RNA levels across all normal tissue types examined. We then developed a score to quantify tumor specificity of gene expression. Compared to previously reported TAA genes, those we identified exhibited substantially greater tumor specificity. Seven of these genes demonstrated consistently elevated expression in at least five tumor types and minimal expression across all non-immune-privileged normal tissues. To assess Human Leukocyte Antigen (HLA) class I presentation potential of the multi-cancer-associated genes, we computationally predicted the binding affinities between the most common class I HLA alleles and all possible wild-type TAA epitopes of 8–11 amino acids arising from those genes. We then applied rigorous filtering criteria to prioritize the most promising multi-cancer TAA peptide candidates and evaluated their HLA binding and cell-surface presentation using T2 and immunopeptidome analysis. Our results highlight new potential targets for multi-cancer immunotherapies.

## Introduction

The immune system recognizes two main categories of antigenic molecules that can elicit antitumor immune responses: tumor-specific antigens (TSAs) and tumor-associated antigens (TAAs). TSAs, also known as neoantigens, arise from somatic mutations (including single-nucleotide mutations, insertions or deletions, gene fusions and aberrant splicing) that alter the amino acid composition of a protein, or from the expression of viral oncoproteins (1, 2). When presented on the cell surface by Human Leukocyte Antigen (HLA) molecules, TSA peptides can be recognized by T cells, which bind to the HLA-peptide complex and activate immune responses against tumor cells displaying that complex.

TSA-based vaccines in combination with immune checkpoint inhibitors have been used to treat patients with various types of cancers in a highly targeted manner (3–8). However, due to the private (unique) nature of somatic mutations, such personalized vaccines typically must be designed on a per-patient basis (1, 2), incurring high costs and requiring custom bioinformatic analyses that are currently impractical for widespread adoption in patient-care settings. Although TSA-based vaccines targeting recurrent somatic mutations have been developed (9), their utility remains limited to patients who harbor the specific mutations and express them at sufficient levels. In addition, “cold” tumors with a lower mutational burden offer fewer therapeutic targets for TSA vaccines, restricting the number of patients who could benefit from this approach (2, 9).

In contrast to TSAs, TAAs are non-mutated peptides expressed in cancer tissue at abnormally high levels but in normal tissue either at low levels, during only certain developmental stages, or in a highly tissue-restricted manner (e.g. cancer/testis antigens) (2, 10, 11). Like TSAs, TAAs can be presented on cell surface HLA molecules and induce T-cell responses (12–15). Although their susceptibility to immune tolerance mechanisms and their lack of strict tumor specificity may provoke less-robust T-cell activation or induce off-target toxicity in response to vaccination, these concerns can be mitigated to some extent by using immunostimulatory adjuvants, administering multiple doses of TAA-based vaccines, or modifying the drug delivery system (9, 11, 14, 16–18). In addition, other therapeutic modalities offer opportunities to target tumor tissue TAAs in a highly specific manner. For example, low-affinity chimeric antigen receptor (CAR-T) therapy can distinguish high-expressing (tumor) tissue from low-expressing (normal) tissue (19, 20), and dual-receptor CAR-T requires two different highly expressed antigens for activation (21, 22).

The shared nature of TAAs across patients makes them attractive targets for readily deployable off-the-shelf immunotherapies. Multiple studies have documented the ability of TAA vaccines to induce antitumor immune responses. For example, cancer vaccines have been developed to target ERBB2/HER2, a tyrosine kinase receptor whose overexpression in 15-30% of breast cancer patients is associated with worse overall survival (23, 24). Typically administered in conjunction with immunostimulatory adjuvants such as Granulocyte-macrophage colony-stimulating factor (GM-CSF) or with monoclonal antibodies (e.g. trastuzumab), HER2 peptide vaccines contain epitopes derived from wild-type HER2 that activate T-cell responses against tumor, with clinical benefits reported in multiple studies (24–33). A recent phase I trial assessed a TAA-based vaccine targeting another protein overexpressed in breast cancer, folate receptor alpha (FRα) (34). This peptide vaccine, containing multiple epitopes from wild-type FRα, induced durable T-cell immunity to FRα in a cohort of breast and ovarian cancer patients and showed a good toxicity profile, despite low-level expression of FRα in normal kidney and choroid plexus tissues.

Beyond breast cancer, other cancer types have proven amenable to TAA-based immunotherapies. Several studies in glioblastoma patients have demonstrated favorable T-cell responses after immunizations with vaccines containing TAA peptides either alone or in combination with TSA peptides (35–37). In a small study of 5 lung cancer patients and 5 glioma patients, TAA-specific T-cell responses were observed in patients given dendritic cell vaccines loaded with TAAs highly expressed in tumor (38). Likewise, an RNA-based vaccine targeting four TAAs in melanoma patients showed encouraging results in phase I trials, with strong T-cell activation and tumor shrinkage reported (39). Finally, mRNA vaccines targeting TAAs overexpressed in prostate cancer, in combination with an immune-promoting adjuvant, bolstered T-cell activation against tumor cells in murine models (40).

Previous studies investigating TAAs have largely focused on a small number of antigens and a single cancer type. While some recent studies have identified genes with high cancer-associated expression in large cohorts (41–45), to our knowledge none have identified genes consistently overexpressed across multiple cancer types and comprehensively predicted the HLA class I epitopes arising from those genes. Here, we report a set of protein-coding genes that are aberrantly expressed in multiple solid tumor types in the harmonized TCGA TARGET GTex dataset (46), with minimal expression across all normal tissues assayed. Using a scoring-based approach, we found that these TAA genes demonstrated greater tumor specificity than previously reported TAA genes. We then predicted the presentation potential of all possible wild-type epitopes of 8–11 amino acids in length originating from the overexpressed genes using our previously published REAL-neo pipeline (47), and we identified the top candidate peptides with binding potential to the most common HLAs in TCGA. Finally, we validated the HLA binding and cell-surface presentation of our top TAA candidates using binding assays and immunoprecipitation of HLA-peptide complexes followed by mass spectrometry analysis. Our study uncovers promising multi-cancer therapeutic targets for immunotherapies with broad treatment potential.

## Materials and methods

**Figure 1:**
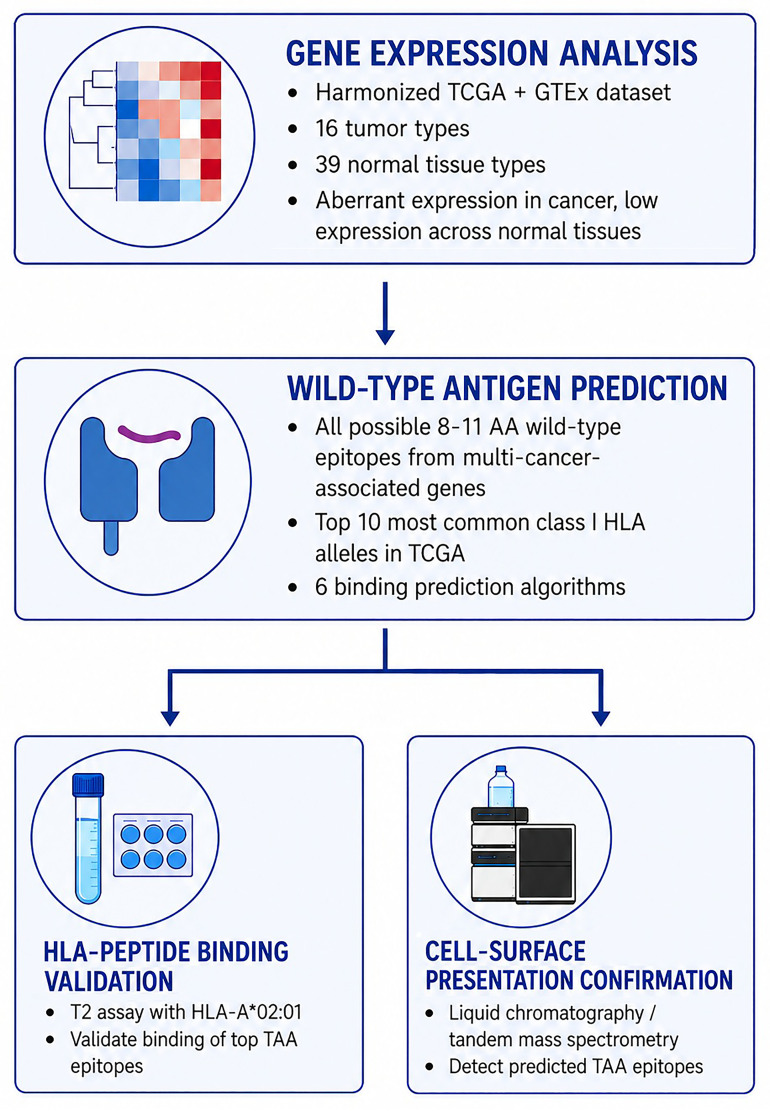
Overview of study design. The TCGA + GTEx dataset was mined for genes aberrantly expressed in multiple cancer types and minimally expressed across normal tissues. All possible class I wild-type antigens from the identified multi-cancer-associated genes were evaluated for binding to the most common class I HLAs using six prediction algorithms. Experimental confirmation of multiple predicted epitopes was performed using two assays. First, a T2 assay measured binding between a subset of top epitopes and HLA-A*02:01. Second, liquid chromatography–tandem mass spectrometry was used to identify predicted epitopes bound to the cell surface of multiple cancer cell lines.

An overview of our methodology is provided in **Figure 1**.

### Gene expression dataset

The harmonized expression data (46) from The Cancer Genome Atlas (TCGA) (48) and the Genotype-Tissue Expression Consortium (GTEx) (49) were downloaded from the UCSC Xena Browser (https://xenabrowser.net) (50). For cancer samples, we focused on the 16 TCGA solid tumor types with at least 10 corresponding normal tissue samples: bladder urothelial carcinoma (BLCA), breast invasive carcinoma (BRCA), colon adenocarcinoma (COAD), esophageal carcinoma (ESCA), head and neck squamous cell carcinoma (HNSC), kidney chromophobe (KICH), kidney renal clear cell carcinoma (KIRC), kidney renal papillary cell carcinoma (KIRP), liver hepatocellular carcinoma (LIHC), lung adenocarcinoma (LUAD), lung squamous cell carcinoma (LUSC), pancreatic adenocarcinoma (PRAD), rectum adenocarcinoma (READ), stomach adenocarcinoma (STAD), thyroid carcinoma (THCA), and uterine corpus endometrial carcinoma (UCEC) (**Supplementary Figure 1**). For normal samples, we included the 7,402 GTEx samples in the harmonized dataset across 51 tissues (39 tissues after consolidation of brain into a single category; cell lines excluded), as well as the 16 normal tissue types in TCGA corresponding to the cancers listed above. No randomization or blinding was performed. Following identification of top candidate TAA genes, as described in the Results, a validation step assessed their expression in benign tissues and other anatomic sites from the Chan Zuckerberg CellxGene Discover database (51) at single-cell resolution.

### Identification and selection of HLA alleles

Optitype version 1.3.1 was used to identify HLA alleles in the primary tumor RNA data from all 33 TCGA cancer types. The 10 most frequently observed HLAs in TCGA that were compatible with all six HLA-peptide binding prediction algorithms (see description below) are shown in **Supplementary Table 1**.

### Generation of wild-type proteome

The amino acid sequences of all proteins were downloaded from the Consensus Coding Sequence Database (CCDS) version 22. To obtain all possible epitopes, sliding windows of length 8, 9, 10 and 11 amino acids were tiled along the sequence of each cancer-associated gene we identified.

### Protein topology prediction

Topology of the identified cancer-related genes was predicted using DeepTMHMM (52), which leverages deep learning models to label each residue as intracellular/cytosolic, extracellular/extraluminal, alpha membrane, beta membrane, signal peptide or periplasm.

### Peptide-HLA binding prediction

Prediction of peptide-HLA binding affinity was carried out using the REAL-neo workflow (Ren 2020). Specifically, the following tools were used to estimate the IC50 values between all possible wild-type epitope k-mers from the identified cancer-related genes and selected class-I HLA alleles: NetMHC (53) V.4, NetMHCpan (54) V.2.8, SMM (55), SMMPMBEC (56), PickPocket (57) V.1.1 and MHCflurry (58) V.2.0.1. All six tools were initially trained by the developers on experimentally measured HLA-peptide binding affinities from The Immune Epitope Database (IEDB): http://tools.iedb.org/mhci/download/. In our dataset, each algorithm was used to predict binding between the peptide k-mers from selected genes and the 10 common HLAs that were compatible with all 6 tools (tool-specific HLAs, from additional training datasets, were ignored). Peptide-HLA pairs with IC50< 500 nM across all six tools were considered high-confidence putative binders.

### BLAST analysis

The blastp algorithm of the Basic Local Alignment Search Tool (BLAST) version 2.16.0 was used to identify exact matches between the identified wild-type epitope candidates and all human proteins belonging to the GRCH38 assembly in the BLAST refseq_protein database.

### Tumor specificity score

We developed a TAA score to compare the tumor specificity of known TAA genes to that of the identified multi-cancer TAA genes. The score rewards genes with aberrantly high expression in a given tumor tissue type relative to the corresponding normal, rewards genes with expression below the detection threshold in normal, and penalizes genes in a monotonic manner as the number of normal tissue types expressing that gene increases. It is calculated as follows:


$${\text{TAA}}_{\text{gene,cancer}}=\log2(\log2{\text{FC}}_{\text{gene,cancer}}\cdot {\text{TumorDelta}}_{\text{gene,cancer}}\cdot {\text{NormalDelta}}_{\text{gene,cancer}}\cdot {\text{GTExPenalty}}_{\text{gene}})$$

The log2 fold change from the differential expression analysis measures the difference in expression between tumor and normal tissue for a particular gene. TumorDelta, defined as max(E_tumor_ – E_cutoff_, 0), measures the distance of median expression in tumor above the expression-detection threshold, with a value of 0 given to genes showing expression below that threshold. NormalDelta, defined as max(E_cutoff_ − max(E_normal_, 0), 0), is the distance of median expression in normal tissue below the detection threshold, with negative values clipped to zero to avoid subtracting a negative. Finally, GTExPenalty_gene_, defined as 1/(1 + Count_GTEx_), divides 1 by the number of normal tissue types in GTEx with median expression above the detection threshold. The log2 transformation compresses extreme values and stabilizes variance.

### T2 binding assay

The T2 assay indirectly infers binding between peptides and HLA molecules by measuring peptide-dependent stabilization of cell-surface HLA expression on TAP-deficient T2 cells. All TAA peptides tested in the assay were synthesized by GenScript Biotech (Piscataway, New Jersey) with a minimum purity of 85.0%. The FLU peptide GILGFVFTL, a known HLA-A*02:01 binder, and peptide HPVGEADYF, a known HLA-B*35:01 binder, served as positive and negative controls, respectively. T2 cells (ATCC, CRL-1992) were grown in Iscove Modified Dulbecco’s Medium supplemented with 20% Fetal Bovine Serum. Prior to the assay, T2 cells were washed twice with PBS and plated in serum-free IMDM at a density of 250,000 cells per well in a 12-well plate. Cells were then incubated with the control or TAA peptides at 25uM for 16 hours at 26 °C under 5% CO2. Following incubation, cells were collected, washed twice with PBS, and resuspended in 100 µl of FACS buffer. Next, cells were treated with TruStain FcX FC receptor block (BioLegend, San Diego, California) to prevent nonspecific binding and then stained with the viability dye sytox-red (Thermofisher) and anti-HLA-A2 BV421 (BD Biosciences). Cells were gated on singlets and viability prior to acquisition of HLA-A2 signal. Events were collected on an Attune NxT flow cytometer.

### Immunopeptidome analysis

Class I immunopeptides were isolated from tumor cell lysates and analyzed by liquid chromatography–tandem mass spectrometry. The cell lines present in this study were obtained from the American Type Culture Collection (ATCC). Detailed description of cell line selection, sample preparation, mass-spectrometric settings and bioinformatic processing is provided in the **Supplementary Methods**.

### Off-target effects screening

For screening at the gene level, the Chan Zuckerberg CellxGene Discover database (51) was queried to evaluate expression of the top candidate TAA genes at single-cell resolution in hematopoietic cell types, which comprise a high-risk compartment for therapeutic targeting due to their immune functions and systemic distribution. Only the cell types that each comprised at least 1% of total blood cells were analyzed. For screening at the peptide level, the HLA Ligand Atlas (59) was interrogated for any peptides presented in benign tissues that overlapped with the candidate antigenic peptides nominated by our pipeline.

## Results

### Identification of genes aberrantly expressed in tumor

**Figure 2:**
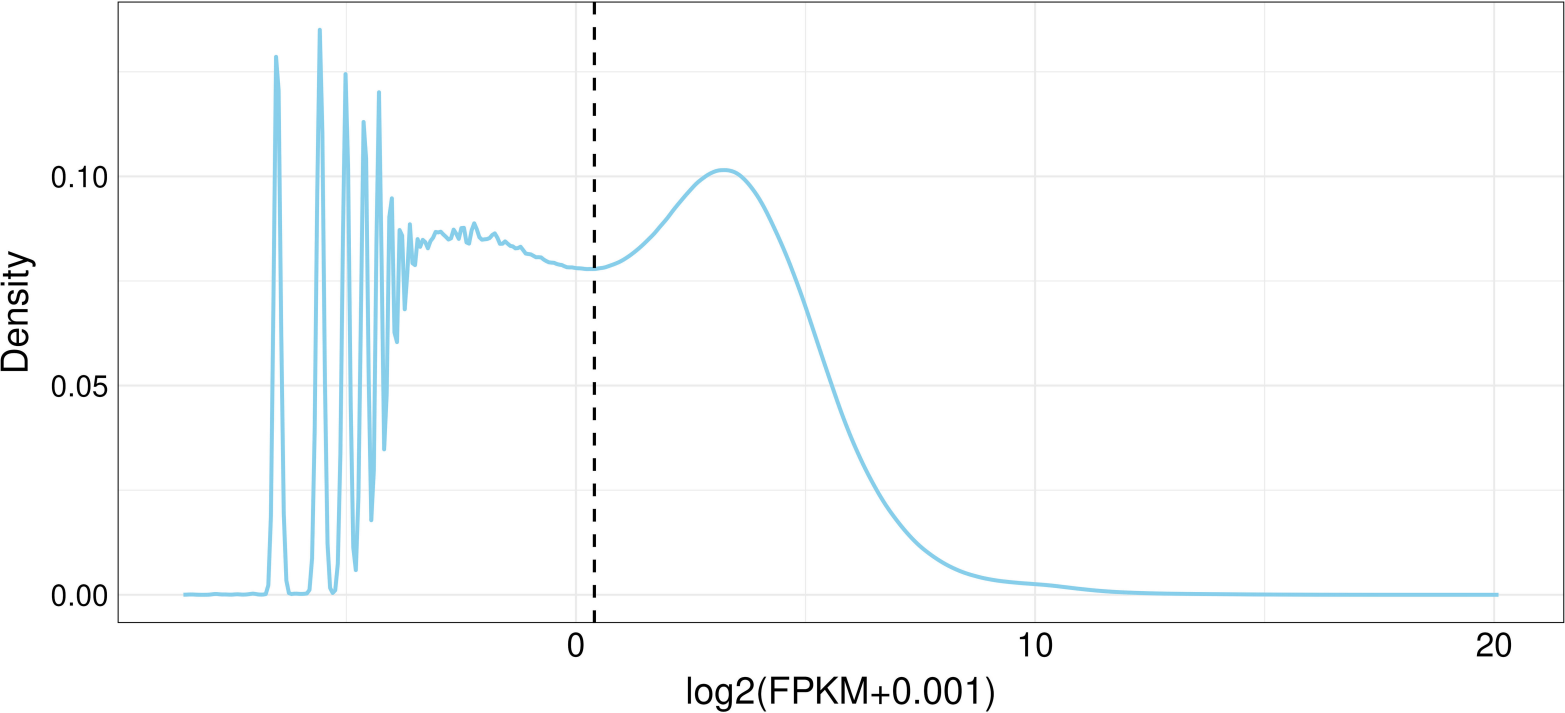
Distribution of log2(FPKM + 0.001) across all genes for 6,353 primary tumor samples from TCGA and 8,801 normal tissue samples (679 from TCGA and 7,402 from GTEx) in the harmonized dataset. The dashed vertical line at 0.4009 denotes the boundary between expressed and unexpressed genes. For visualization purposes, the lowest value was excluded from the density plot.

We analyzed the log2-transformed FPKM of all genes (n=60,498) in all samples (n=14,434) as described in the Methods and identified the local minimum of the bimodal distribution as the expression detection threshold (log2 (FPKM + 0.001) = 0.4009, corresponding to untransformed FPKM of 1.32) (**Figure 2**).

For each of the 16 tissue types from The Cancer Genome Atlas (TCGA) listed in the Methods, genes overexpressed in tumor were identified using the following strategy. First, differential gene expression analysis between the tumor and normal samples was carried out using Limma (60), and protein-coding genes with an adjusted p-value< 0.05 and a log2 fold change > 1 between tumor and normal were retained. Next, to identify genes expressed in tumor but not appreciably expressed in the corresponding normal samples, the differentially expressed genes with median log2(FPKM + 0.001) above the 0.4009 detection cutoff across tumor samples and below 0.4009 across the corresponding normal samples were retained for further analysis. These steps produced, for each TCGA cancer type, a set of differentially expressed genes with aberrantly high expression in tumor and low or negligible expression in the corresponding normal tissue.

**Figure 3:**
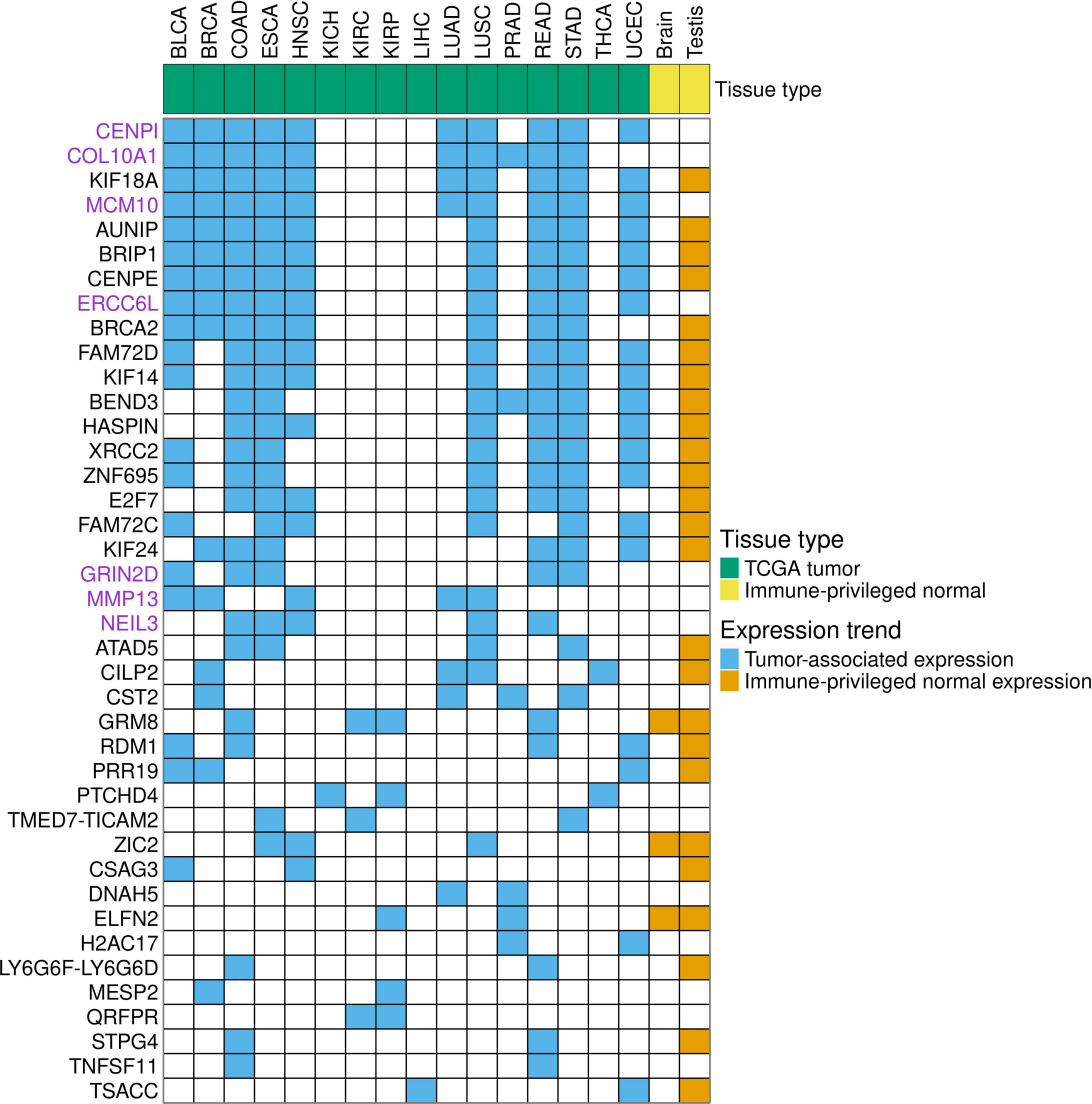
Heatmap of 40 genes with tumor-associated expression detected in at least two TCGA cancer types and with overall negligible expression in all non-immune-privileged normal tissue types in GTEx and TCGA. The annotation bar at the top indicates tissue type, with TCGA tumor type and immune-privileged normal tissue types denoted by green and yellow squares, respectively. For TCGA tumors, genes highlighted with blue squares passed all filters for a given cancer type: significant differential expression and a log2 fold-change greater than 1 between tumor and normal; median log2(FPKM + 0.001) above the expression cutoff in tumor; and median log2(FPKM + 0.001) below the expression cutoff in normal. For GTEx immune-privileged tissues, genes highlighted with goldenrod boxes had median expression above the cutoff. Genes depicted in purple text passed filters in at least 5 cancer types and also showed overall negligible expression in immune-privileged normal tissue.

Next, to pinpoint which of these genes showed expression below the detection threshold across all normal tissue types, we filtered out genes with median expression exceeding the cutoff of 0.4009 in any of the 16 TCGA normal tissue types and in any non-immune-privileged (61, 62) tissue types (i.e. all tissues except brain and testis) among the Genotype-Tissue Expression (GTEx) samples. We identified 94 such genes with consistently elevated expression in at least one cancer type and minimal expression in all non-immune-privileged normal tissues (**Supplementary Table 2**). Of these 94 genes, 40 showed elevated median expression in at least 2 cancer types (**Figure 3**).

### Comparison to known TAA genes

Previous studies have identified antigenic wild-type peptides from multiple genes and gene families with aberrant tumor-associated expression, including the cancer-germline genes and gene families *MAGE, GAGE/XAGE*, *SAGE*, *CTAG/NY-ESO-1*, *SSX*, *CCNA/Cyclin A, CCNB/Cyclin B, KLC1,KMHN1/CCDC110*, *SPA17* and *PRAME* (reviewed in (14, 63)). In addition, tumor-associated genes targeted by Chimeric antigen receptor (CAR-T) approaches include *EGFR*, *B7H3*/*CD276*, *ROR1*, *MSLN*, *CD70*, *CD44*, *CD133*/*PROM1*, *GD2*/*B4GALNT1*, *EPCAM*, *CEA*/*CEACAM6*, *CD147*/*BSG*, *NKG2DL*/*KLRK1*, *DR5*/*TNFRSF10B*, *CD70*, *CAIX*/*CA9*, *VEGFR2*/*KDR*, *ROR2*, *MMP2*, *PDL1*/*CD274*, *DLL3*, *CLDN18*, *CD276*, *CD44*, *PSCA*, *ALPPL2*, *CLDN6*, *PSMA*/*FOLH1* and *Fra*/*FOLR1* (reviewed in (64)). Other genes with documented overexpression in cancer that have been suppressed by immunotherapies include *WT1* (65, 66), *AFP* (67, 68), *GPC3* (69, 70), *BIRC5/Survivin* (71), *MUC1* (72–74), *FRα* (34), *ERBB2/HER2* (55) and *CEACAM6* (75).

We surveyed these known TAA genes in the harmonized TCGA and GTEx dataset (**Supplementary Table 3**; **Supplementary Figure 2**) and found that 5 overlapped with the 94 genes we independently identified by our stringent filtering approach described above: *ALPG, SSX1*, *XAGE1A* and the *MAGE* gene family members *MAGE-A3* and *MAGE-A4*; however, none of these 5 passed all filters in more than one cancer type. Other known TAA genes, while generally highly expressed in multiple cancer types, showed moderate or even high expression in several non-immune-privileged normal tissues: *B4GALNT1*, *BIRC5, BSG*, *CA9*, *CD44*, *CD70*, *CD274*, *CD276*, *CLDN18*, *CCNB1*,*CEACAM6, EGFR*, *EPCAM*, *ERBB2*, *FOLH1*, *FOLR1*, *GPC3*, *KDR*, *KLC1*, *KLRK1*, *MMP2*, *MSLN*, *MUC1*, *PROM1*, *PSCA*, *ROR1*, *ROR2*, *SPA17* and *TNFRSF10B*. Some genes were expressed predominantly in testis but also in several cancer and normal tissues: *CCDC110*, *CCNA1*, *PRAME* and *SSX1*. Median expression of *CTAG1A, SAGE1* and *GAGE1* fell above the expression threshold only in testis, although patient-to-patient variability was widespread. Conversely, *AFP*, *ANKRD30A* and *CLDN6* exhibited relatively low expression in most cancer and normal tissue types. Notably, only normal tissues expressed *WT1* and *DLL3* at appreciable levels overall, but this observation could stem from the types of tumors included in TCGA. For example, small cell lung cancers, neuroendocrine tumors and Merkel cell carcinomas, known to overexpress *DLL3* (76, 77), were not profiled in TCGA.

**Figure 4:**
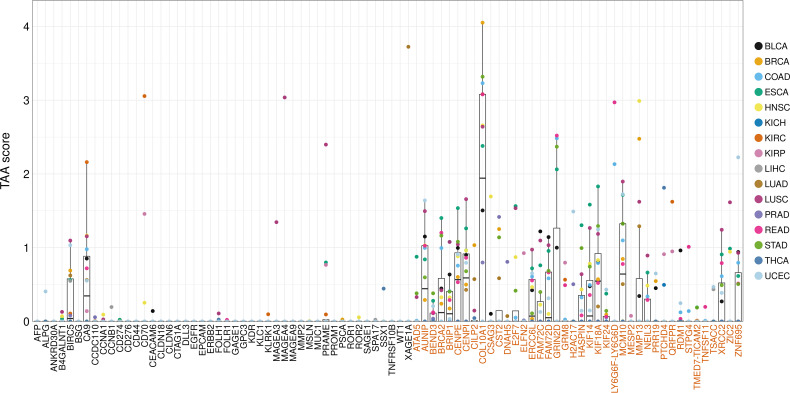
TAA score comparison between previously reported TAA genes and newly identified multi-cancer TAA genes among 16 TCGA cancer types. The score, calculated for each gene on a per-cancer basis, quantifies the specificity of tumor expression by rewarding genes with large differences between tumor and normal expression for a given tissue type and penalizing genes expressed in one or more other normal tissue types. The 46 previously reported TAA genes are denoted by black text, while the 40 multi-cancer TAA genes are denoted by orange text. In general, the multi-cancer TAA genes reached substantially higher TAA scores across a greater number of cancer types due to their high expression in tumor tissue and low expression across normal tissues. In contrast, the previously reported TAA genes showed overall higher expression across multiple normal tissue types and therefore have lower TAA scores.

To compare the tumor specificity of the known TAA genes to that of the 40 genes identified by our approach, we calculated a TAA score for each gene on a per-cancer basis (**Supplementary Table 2****;** **Supplementary Table 3**). The score rewards genes that have aberrantly high expression in a given TCGA tumor tissue and minimal expression in the corresponding TCGA normal tissue and penalizes genes with detectable expression in other normal, non-immune-privileged GTEx tissue types, as described in the Methods. As shown in **Figure 4**, the 40 multi-cancer TAA genes we identified showed substantially greater tumor specificity than the previously reported TAA genes. While several known TAA genes sporadically achieved relatively high scores in a small number of cancer types, the newly reported TAA genes displayed consistently high scores across multiple tumor types, with 9 cancers showing differences in the distribution of TAA score between the two sets of TAA genes (Wilcoxon rank-sum test; adjusted p-value< 0.05) (**Supplementary Table 4**). These results indicate that the widely recognized TAA genes have overall lower tumor specificity and greater patient-to-patient variability compared to the multi-cancer TAA genes identified by our computational approach.

### Selection of top candidate TAA genes

**Figure 5:**
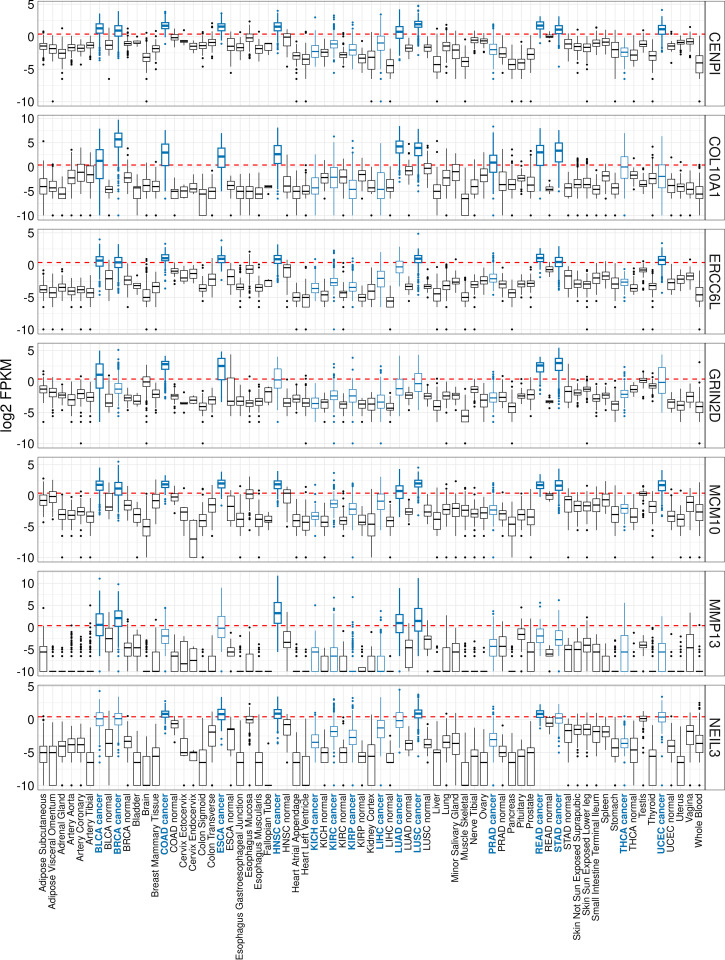
Distribution of log2(FPKM + 0.001) for seven genes with tumor-associated median expression in at least five cancer types and overall low expression across all normal tissues examined. Blue color indicates tumor tissue and black color indicates normal tissue. Bold face denotes median expression above the detection threshold, which is demarcated by the red dashed line.

To select potential sources of multi-cancer TAAs, we focused on the tumor-related genes that were expressed in at least five cancer types and that fell below the median expression cutoff in both non-immune-privileged and immune-privileged normal tissues: *Centromere protein 1* (*CENPI*) (CCDS14479.1), *Collagen type X alpha-1 chain* (*COL10A1*) (CCDS5105.1), *Excision repair cross-complementation group 6 like* (*ERCC6L*) (CCDS35329.1), *Glutamate ionotropic receptor NMDA type subunit 2D* (*GRIN2D*) (CCDS12719.1), *Minichromosome maintenance protein 10* (*MCM10*) (CCDS7095.1), *Matrix metalloproteinase-13* (*MMP13*) (CCDS8324.1) and *Nei endonuclease VIII-like 3* (*NEIL3*) (CCDS3828.1) (**Figure 3**, genes in purple text; **Figure 5**).

**Figure 6:**
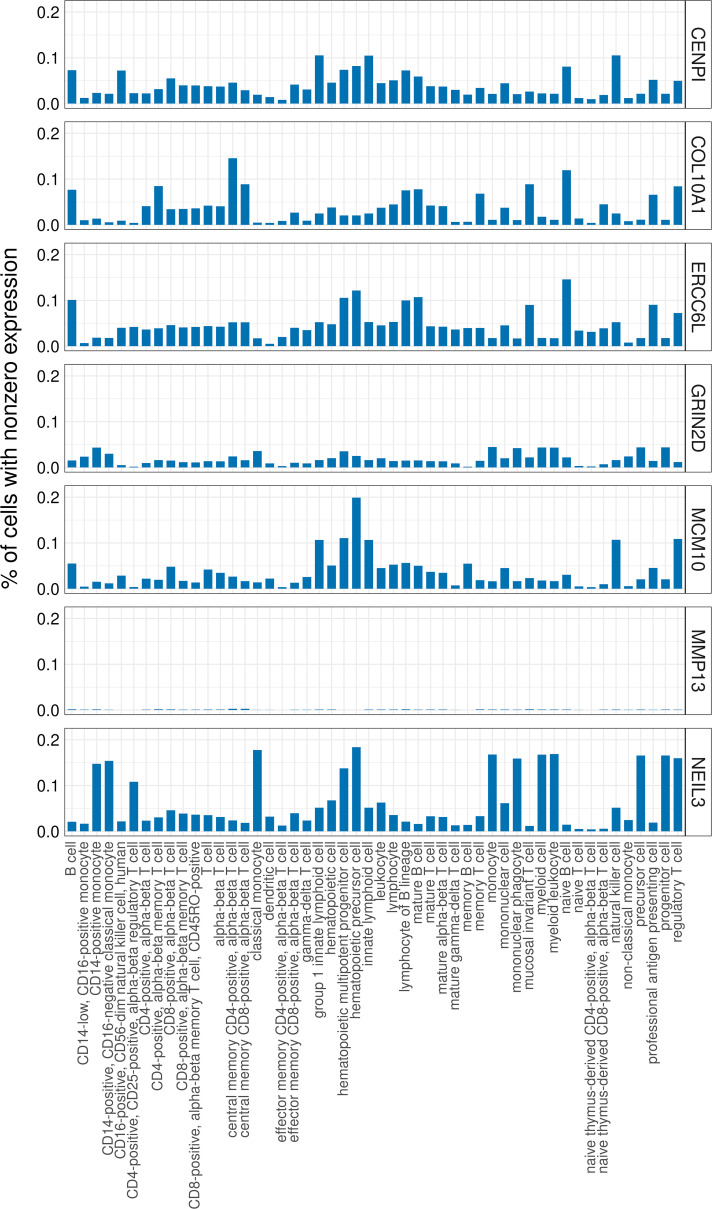
Single-cell RNA sequencing expression proportions for 7 TAA genes across 50 hematopoietic cell types in Chan Zuckerberg CELLxGENE Discover. The percentage of cells with nonzero expression was calculated for each cell type as the number of cells with at least one transcript-supporting read detected for a given gene divided by the total number of cells assigned to that cell type.

To validate the negligible expression in noncancerous specimens observed in TCGA and GTEx, we queried a separate dataset of 63 sample types (including solid tissues, anatomic sites and biological fluids) (51) for the seven top genes at single-cell resolution, using a conservative detection threshold of one transcript-supporting read per cell. Aggregating all cell populations within each specimen type revealed all seven genes expressed in less than 6.6% percent of cells within each sample, with most genes expressed at substantially lower levels, validating the findings from the bulk RNA sequencing analysis (**Supplementary Figure 3**; **Supplementary Table 5**). While none of the seven genes showed appreciable expression in bulk-collected whole blood in GTEx (**Figure 5**), we profiled the same single-cell RNA sequencing data to inspect their expression levels within the 50 individual hematopoietic cell types that each comprised at least one percent of all blood cells. Across all hematopoietic cell populations, the proportion of single cells with detectable expression fell below 0.2% for each of the seven genes (**Supplementary Table 6**; **Figure 6**), suggesting that targeting the genes therapeutically would not inadvertently deplete essential immune cell populations.

### TAA peptides predicted in top multi-cancer tumor-associated genes

**Figure 7:**
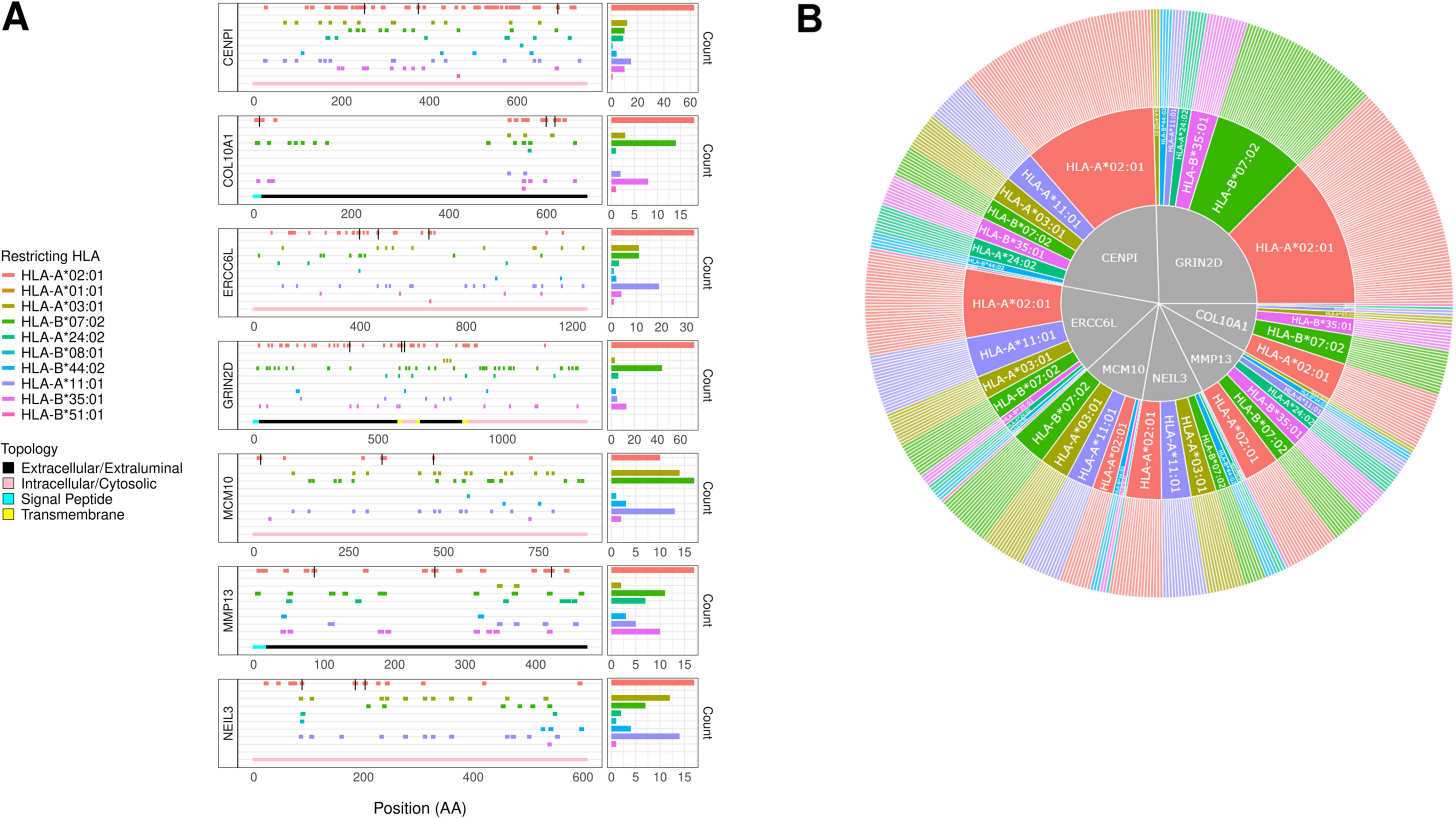
**(A)** Amino acid positions and epitope counts per HLA for the 577 candidate TAA-epitope pairs within the seven pan-cancer-associated genes. The colored rectangles highlight, for each of the 10 most common HLAs in TCGA, the position of each epitope within the full-length protein sequences. The black vertical tick marks denote the HLA-A*02:01 binders selected for the T2 assay. The bar plots indicate the number of epitopes predicted to bind to each HLA. No HLA-B*51:01 binders passed all filters for this set of genes. The solid line segments at the bottom of each panel indicate the predicted topology of the protein sequences at each position. **(B)** Sunburst plot of the 577 candidate TAAs organized by gene and HLA allele. Each segment represents one peptide.

We next assessed the potential of our 7 rigorously defined tumor-associated genes to produce TAA epitopes. To identify TAA peptides compatible with a large cross-spectrum of the population, we predicted the binding affinity between the 10 most common HLA alleles in TCGA (**Supplementary Table 1**), which collectively cover 89.77% of TCGA samples, and the 381,595 possible wild-type peptide k-mers of length 8–11 aa originating from the 7 selected genes. To focus on the most high-confidence candidate TAAs, we retained only the 577 epitope-HLA pairs with binding affinity (IC50)< 500 nM across all 6 prediction algorithms (**Figures 7A, B**). The epitopes were distributed throughout each protein sequence, except those from COL10A1, whose antigenic sequences were concentrated near the N-terminus and C-terminus of the protein. Consistent with the known localization of the proteins, DeepTMHMM predicted intracellular topologies for CENPI, ERCC6L, MCM10 and NEIL3; extracellular topologies for COL10A1 and MMP13; and both intracellular and extracellular topologies for the transmembrane protein GRIN2D (**Figure 7**). In general, the putative HLA-A*02:01 binders outnumbered binders to each of the other HLAs, perhaps due to the proportionally larger HLA-A*02:01 dataset in the Immune Epitope Database (IEDB) used to train the prediction algorithms (**Supplementary Table 1**) and to the well-established broad repertoire of this allele (78).

Consolidating the peptides binding to multiple HLA alleles and the shorter sequences nested within longer sequences yielded 422 unique candidate TAA epitopes (**Supplementary Table 7**). The blastp algorithm detected no exact matches between any other genes in the GRCh38 assembly and the TAA epitopes of CENPI, COL10A1, ERCC6L, MCM10, MMP13 and NEIL3. Eighteen GRIN2D epitopes matched sequences in the closely related proteins GRIN2B, GRIN2C and GRIN3D (**Supplementary Table 7**). These results underscore the high specificity of the putative TAAs, potentially reducing the risk of off-target effects in therapeutic settings.

### Validation of HLA presentation of TAA-derived peptides using T2 binding assay

**Figure 8:**
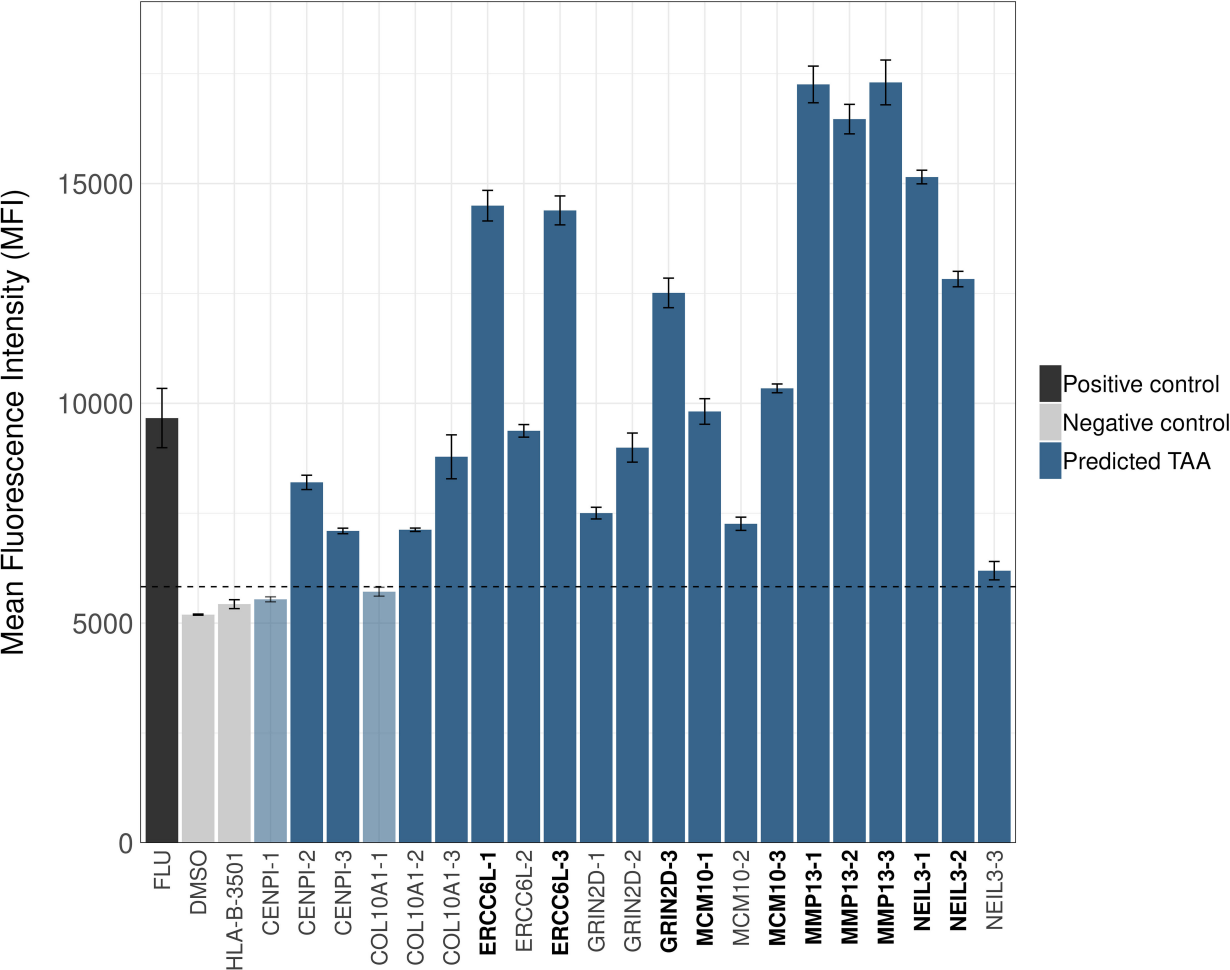
T2 validation of binding between HLA-A*02:01 and 21 selected candidate TAA peptides. Mean fluorescence intensity (MFI) is shown on the y-axis, with error bars indicating the standard error of 2–3 replicates per peptide. Nineteen of the 21 tested candidate TAAs bound to HLA-A*02:01 (dark blue bars), with 10 TAAs showing stronger binding than the positive control peptide from the Influenza virus (bold text). The negative controls consisted of a DMSO vehicle-only assay and peptide HPVGEADYF, an HLA-B*35:01 binder. The dashed horizontal line represents the binding threshold, defined as three standard deviations above the mean of negative controls.

*In vitro* T2 binding assays were performed for validation of class-I HLA presentation of the 21 top candidate TAAs out of the 577 identified by our pipeline. Specifically, three TAA epitopes predicted to bind to HLA-A*02:01 were selected for testing from each gene, with priority given to peptides that had the lowest predicted median IC50 values and that, where applicable, contained shorter nested peptides likewise predicted as strong binders (**Figure 7A**, tick marks; **Figure 8**; **Supplementary Table 7**). Of the 21 peptides tested experimentally, HLA binding was confirmed for 19 (90.5%), 10 of which had higher binding affinities than the positive control influenza virus peptide (**Figure 8**). These results highlight the accuracy of the computational predictions for HLA-peptide binding.

### Confirmation of TAA cell-surface presentation in tumor

To further validate the presentation of the TAA epitopes, an unbiased mass-spectrometry analysis was carried out to identify HLA class-I-bound peptides presented on the surface of cancer cells. Specifically, five CCLE cell lines were selected (three from lung adenocarcinoma, one from lung squamous cell carcinoma and one from breast adenocarcinoma; **Supplementary Table 8**) with expression patterns that mirrored those observed in the TCGA-GTEx analysis. In total, nine of the computationally predicted TAA epitopes listed in **Supplementary Table 7** were detected on the cell surface of at least one of the tested cancer cell lines at a stringent FDR threshold of 1%, a result consistent with the sensitivity limitations and high false-negative rate of HLA mass spectrometry (79, 80). The validated epitopes included two from CENPI, two from ERCC6L, one from NEIL3 and four from MCM10 (**Supplementary Table 7**). Of those nine TAA epitopes confirmed by the assay, three overlapped with the set of 19 peptides validated by the T2 binding assay (**Supplementary Table 7**). The HLAs predicted to bind to each of the mass spectrometry-validated epitopes matched the HLAs expressed by their respective cell lines, with two exceptions: the predicted HLA-A*11:01 binder KVCDIYINY and the predicted HLA-B*07:02 binder APKKKIQTTL were detected on the surface of NCI-H441 and HCC193, respectively, which lacked those HLA alleles; the peptides instead likely bound to different expressed HLAs on which we did not make predictions. These results provided proof of principle that our computational approach could identify TAAs bound to HLA on the cell surface.

### Assessment of TAA presentation in benign tissues

Because some TAA peptides may exhibit low-level presentation in benign tissues, we queried the immunopeptidome data of the HLA Ligand Atlas (59) to identify any overlap between our 577 candidate TAAs and previously reported HLA ligands. In total, seven candidate peptides showed some degree of overlap with the Atlas (**Supplementary Table 7**). Four peptides had exact matches: IPASNRLLL (CENPI) detected in cerebellum; KLDEHIAYL (ERCC6L) detected in bone marrow; FPYPYAERL (GRIND2) detected in esophagus; and FLFGEVHKA (MCM10) detected in cerebellum. In addition, two class II peptides from the Atlas fully encompassed class I candidate TAA epitopes: QLLQNIHCLELPSQ (QLLQNIHCL; CENPI) detected in lung and FTRLHDGIADIMISFGIK (RLHDGIADI; MMP13) detected in spleen. While their presence in the Atlas further validated our TAA identification approach, these six peptides may represent suboptimal therapeutic targets due to possible immune tolerance or potential safety issues in vital tissues.

## Discussion

The ability of TAA-based vaccines to induce antitumor immune responses has been widely reported (29, 34, 35, 39, 40, 45). Whereas previous studies have generally focused on single genes and single cancer types, our study is the first to comprehensively evaluate, using both bioinformatic and proteomic approaches, the HLA binding potential of all possible TAAs arising from protein-coding genes with patterns of multi-cancer-associated expression. We prioritized seven such genes aberrantly expressed in multiple solid tumor types and confirmed the HLA binding and cell-surface presentation of a subset of their candidate TAA peptides.

Previous work suggests that these genes represent viable therapeutic targets. Some small-scale studies in single cancer types have directly investigated the immunotherapeutic potential of TAAs targeting three of the genes we prioritized: *CENPI*, *NEIL3* and *GRIN2D*. CENPI helps to facilitate chromosome alignment and segregation during mitosis. Its overexpression in various cancers has been linked to tumor cell proliferation, immune cell infiltration, and worse survival outcomes (81–85). A recent study tested the immunogenicity of wild-type epitopes derived from *CENPI* and several other cancer-associated genes (86). Binding of 5 bioinformatically predicted *CENPI* epitopes to HLA-A*02:01 as well as T-cell induction were confirmed experimentally. Although the study focused on binding potential between only HLA-A*02:01 and 9-mer epitopes and relied on a single binding prediction algorithm, it provided encouraging evidence that *CENPI* could be targeted therapeutically.

The DNA glycosylase NEIL3 promotes genome integrity by repairing intrastrand DNA cross-links and oxidative damage during replication, and its aberrant expression in tumor has been associated with higher mutational burden, cell proliferation and worse survival outcomes in multiple cancers (87–91). In two phase I clinical studies, patients with liver cancer were treated with one of two vaccine cocktails containing wild-type antigens derived from 3 genes, including *NEIL3* (92), that targeted two HLA-A alleles. Although no patients in either study exhibited complete or partial response to the vaccine, 9/18 patients in the first study and 5/14 in the second exhibited stable disease and therefore better survival, suggesting that this gene could represent a viable therapeutic target.

Overexpression of *GRIN2D*, a component of the N-methyl D-aspartate receptor (NMDAR), promotes oncogenesis and correlates with poor prognosis in liver, pancreatic and lung cancers (93, 94). Vaccination against GRIN2D in a murine colon cancer model inhibited tumor growth and angiogenesis, although this approach relied on antibody-mediated action rather than generation of T-cell responses against peptides displayed on the cell surface by MHC (95). Despite their limited scope, these results nevertheless suggest the treatment potential of targeting genes with the expression profiles we defined.

The remaining four genes that we prioritized have, to our knowledge, never been targeted by vaccines or other immunotherapies: *MCM10*, *COL10A*, *MMP13* and *ERCC6L*. MCM10 functions in DNA replication by stabilizing replication forks. A prognostic biomarker linked to tumor mutational burden, it has been implicated in tumorigenesis in multiple cancer types, including breast, liver, colon, brain, prostate and lung (96–99). Aberrant expression of *COL10A1*, a component of the extracellular matrix, occurs in diverse cancer types and activates signaling cascades that promote tumor cell invasion and migration and reduce survival (100–104). Similarly, overexpression of *MMP13*, a key regulator of the extracellular matrix, predicts increased tumor mutational burden, microsatellite instability and worse prognosis in cancer patients (100, 105–107). Its knockdown by the selective inhibitor Cmpd-1 inhibited tumor growth in mouse breast cancer models (108). Finally, the multi-cancer oncogenic properties of overexpressed *ERCC6L*, a DNA helicase involved in chromosome separation during mitosis, have likewise been widely reported (109–114).

The TAA genes and epitopes that we validated experimentally offer multi-cancer potential for adjuvant therapy or for prevention strategies in high-risk populations. For the intracellular TAAs (CENPI, ERCC6L, MCM10 and NEIL3), peptide vaccines are a straightforward therapeutic modality. A targeted panel to compare TAA gene expression between a patient’s tumor and normal tissue would allow the selection of the most appropriate TAA vaccine based on the specificity of gene expression in tumor. For example, Wang et al. assessed the expression profiles of known TAA genes in a group of 5 lung cancer and 5 glioblastoma patients (38). Due to widespread heterogeneity in gene expression, they developed a personalized TAA vaccine for each patient based on the requirement of twofold higher TAA expression in tumor compared to normal. A similar approach could be implemented here. In addition, patient-appropriate TAAs could be combined with patient-specific TSAs into a single vaccine, as in (35).

Other therapeutic modalities could target extracellular TAA genes such as *COL10A1* and *MMP13*. For example, chimeric antigen receptor (CAR-T) therapy engages with cell-surface antigens directly instead of relying on HLA presentation of peptide fragments (64, 115). Although more costly and time-intensive, this approach provides greater precision and speed compared to peptide vaccines, grants more control to avoid off-target toxicity (116), and has demonstrated clinical successes in leukemia and lymphoma (117–119) and ongoing clinical trials in solid tumors (64). To better discriminate high-expressing tumor tissue from low-expressing normal tissue, low-affinity CARs could be designed that are activated only under conditions of high expression (19, 20) or that require the presence of multiple high-expressing antigens (for example, several of the 7 TAA genes identified herein) to trigger activation (21, 22). Another approach for extracellular TAA genes is bi-specific T-cell engager (BiTE) therapy, in which engineered antibodies bind to both a target TAA and the universal T-cell marker CD3, triggering T-cell activation against the TAA independent of T-cell specificity (115). BiTEs directed against TAAs have achieved clinical success. For example, in a phase II clinical trial, patients treated with a HER2-targeting BiTE treatment showed improved survival and increased antitumor responses (120). Similarly, the BiTE therapeutic Tarlatamab, recently FDA approved (121) to target the TAA protein DLL3 in small-cell lung cancer, demonstrated long-lasting objective responses and antitumor activity in phase 1 and phase 2 trials (122, 123).

Several limitations of this study warrant consideration. While our study confirmed HLA binding and presentation of computationally predicted TAA epitopes, further functional validation is required to establish their immunogenicity and therapeutic utility. In addition, multiple tumor-associated genes demonstrated low-level expression in normal tissues, as expected for wild-type transcripts. While the lack of truly tumor-specific expression does not preclude the therapeutic utility of TAA genes (e.g (24, 29, 33, 34)), careful safety assessments are required to ensure no off-target effects from therapeutic products targeting the TAAs, such as vaccines, CAR-T cells, antibody drug conjugates, and bispecific antibodies. These potential off-target effects are particularly relevant if these TAAs are expressed in circulating immune cells. In consideration of these risks, we surveyed single-cell RNA sequencing data and found that fewer than 0.2% of cells from each of 50 hematopoietic cell populations showed any detectable expression of the seven selected multi-cancer TAA genes, suggesting that therapeutics targeting their epitopes pose minimal hazard to the immune system. In addition, we flagged the six epitopes previously identified as HLA ligands in benign tissues in order to de-prioritize peptides with potential off-target effects, particularly in non-immune privileged tissues. While these six epitopes represented suboptimal therapeutic targets, their presence in the HLA Ligand Atlas validated the ability of our pipeline to identify presented peptides. In addition, the T2 and immunopeptidome assays reported here established the HLA binding potential and cell surface presentation of multiple epitopes nominated by our approach, but antigen-specific T-cell assays are required to test their capacity to induce immune responses. Despite these limitations, the TAAs we identified offer broad treatment potential for patients with diverse cancers.

## Data Availability

The datasets presented in this study can be found in online repositories. The names of the repository/repositories and accession number(s) can be found in the article/**Supplementary Material**.
