# Supplementary material for: Identification of tumor-associated antigens with multi-cancer therapeutic potential
Source: Front Immunol. 2026 Jun 5;17:1839980. doi: 10.3389/fimmu.2026.1839980 (PMC13279219; doi:10.3389/fimmu.2026.1839980)
Supplement: Supplementary file 1 [file Table1.docx]

Supplemental material for

**Identification of tumor-associated antigens with multi-cancer therapeutic potential**

Daniel P. Wickland, Erik Jessen, Asha Nair, Brian Necela, Kimberly Lauer, Kiran K. Mangalaparthi, Rex Devasahayam Arokia Balaya, Akhilesh Pandey, Aaron S. Mansfield, Keith L. Knutson, Yan W. Asmann**^*^**

* To whom correspondence should be addressed. Email: [Asmann.Yan@mayo.edu](mailto:Asmann.Yan@mayo.edu)

**This file includes**

Supplemental Table Legends

Supplemental Figure 1

Supplemental Figure 2

Supplemental Figure 3

Supplemental Methods

Supplemental References

**Supplemental Table 1.** Prevalence of the top 10 most common class-I HLA alleles in TCGA that are compatible with all six HLA-peptide binding prediction algorithms. The sizes of the Immune Epitope Database (IEDB) datasets used to train the HLA-peptide binding algorithms are shown for each HLA.

**Supplemental Table 2.** List of 94 genes identified with aberrantly high tumor-associated expression and minimal normal-tissue expression. Included in the table for each gene and cancer tissue type are the log2 fold-change values between tumor and corresponding normal, the median expression of both tumor and normal, the median expression in each GTEx normal tissue, a list of filters passed, and the TAA score and its components.

**Supplemental Table 3.** List of 46 previously identified TAA genes. Included in the table for each gene and cancer tissue type are the log2 fold-change values between tumor and corresponding normal, the median expression of both tumor and normal, the median expression in each GTEx normal tissue, a list of filters passed, and the TAA score and its components.

**Supplemental Table 4**. Single-cell RNA sequencing expression of the top seven multi-cancer TAA genes across benign non-hematopoietic tissues, anatomic sites and fluid-derived samples. For each sample type, all cell populations are aggregated. Included in the table for each gene and sample type are the number of single cells with detectable transcript signal (>1 transcript-supporting read), total number of cells analyzed for the sample type, and percent of cells with detectable expression.

**Supplemental Table 5**. Single-cell RNA sequencing expression of the top seven multi-cancer TAA genes across hematopoietic cell types. Included in the table for each gene and cell type are the number of single cells with detectable transcript signal (>1 transcript-supporting read), total number of cells analyzed for the cell type, and percent of cells with detectable expression. Cell types that each comprised less than 1% of all blood cells are excluded.

**Supplemental Table 6.** List of 422 unique candidate TAA epitopes in the top seven multi-cancer TAA genes: *CENPI*, *COL10A1*, *ERCC6L*, *GRIN2D*, *MCM10*, *MMP13* and *NEIL3*. Included for each epitope are the HLA allele(s) with strong binding predicted; the median predicted IC50 value of the peptide-HLA pair across 6 prediction algorithms; any genes with exact matches elsewhere in the proteome; any nested epitopes including their HLA binding partner and median IC50 value; results of the T2 assay; results of the immunopeptidome assay; and any matches found in the HLA Ligand Atlas of previously detected epitopes in benign tissue.

**
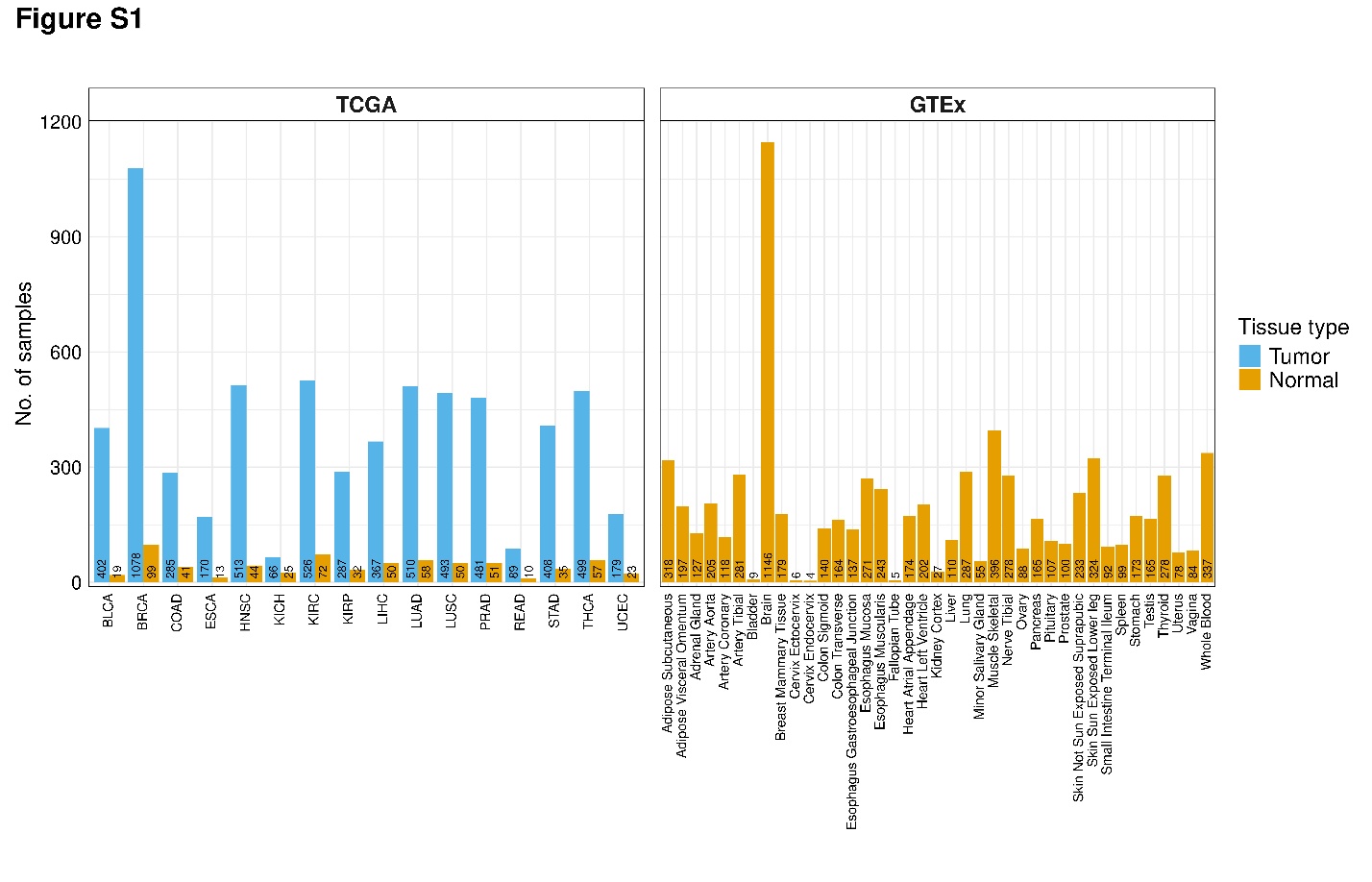
Supplemental Figure 1.** Primary tumor and normal tissue counts among 16 TCGA tissue types with at least 10 normal samples, and normal sample counts among 39 GTEx tissue types. Brain tissues were consolidated into a single category.

*
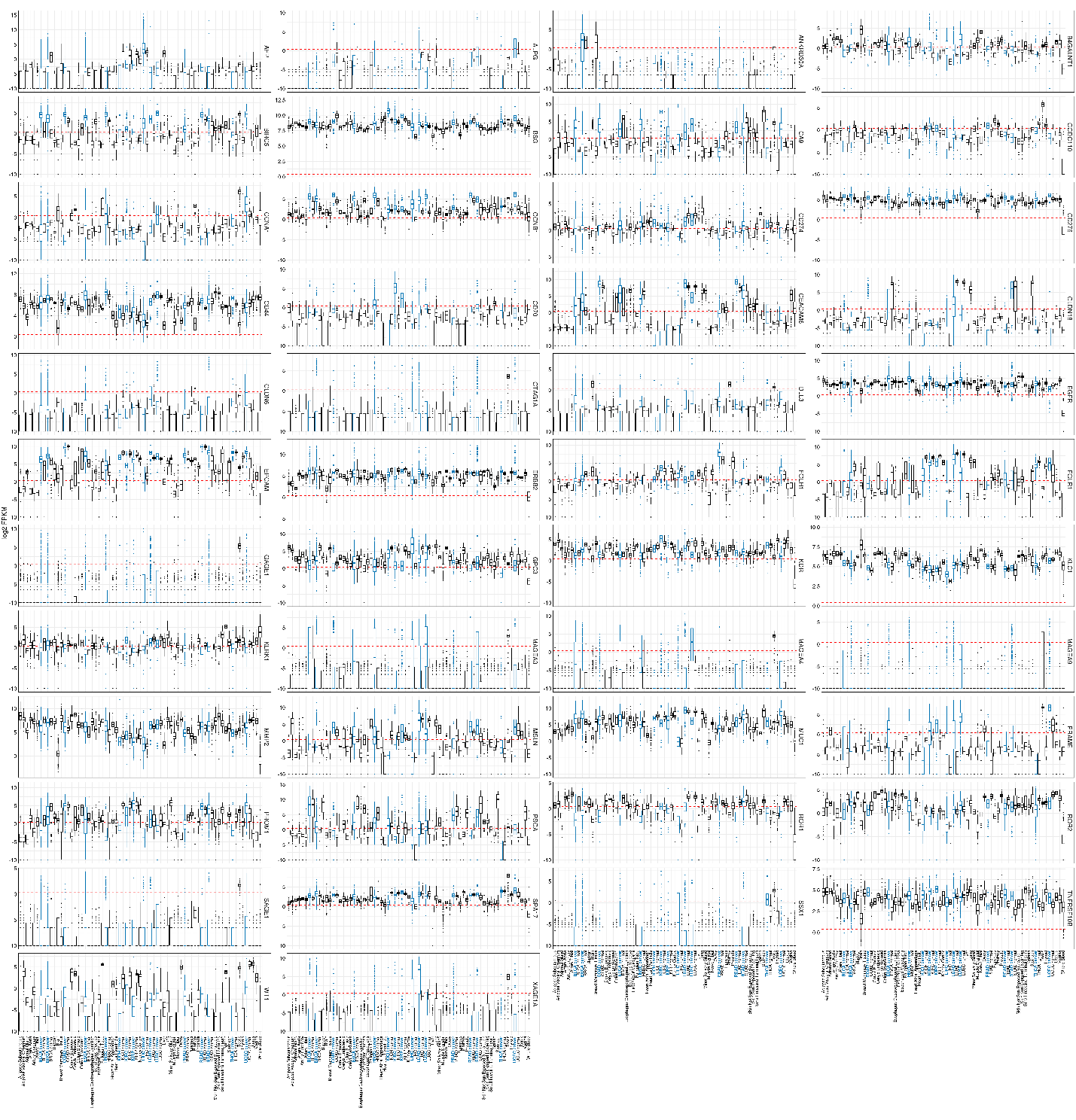
*

**Supplemental Figure 2.** Distribution of log2(FPKM+0.001) for 46 known TAA genes. Blue color indicates tumor tissue and black color indicates normal tissue. Bold face denotes median expression above the detection threshold, which is demarcated by the red dashed line.


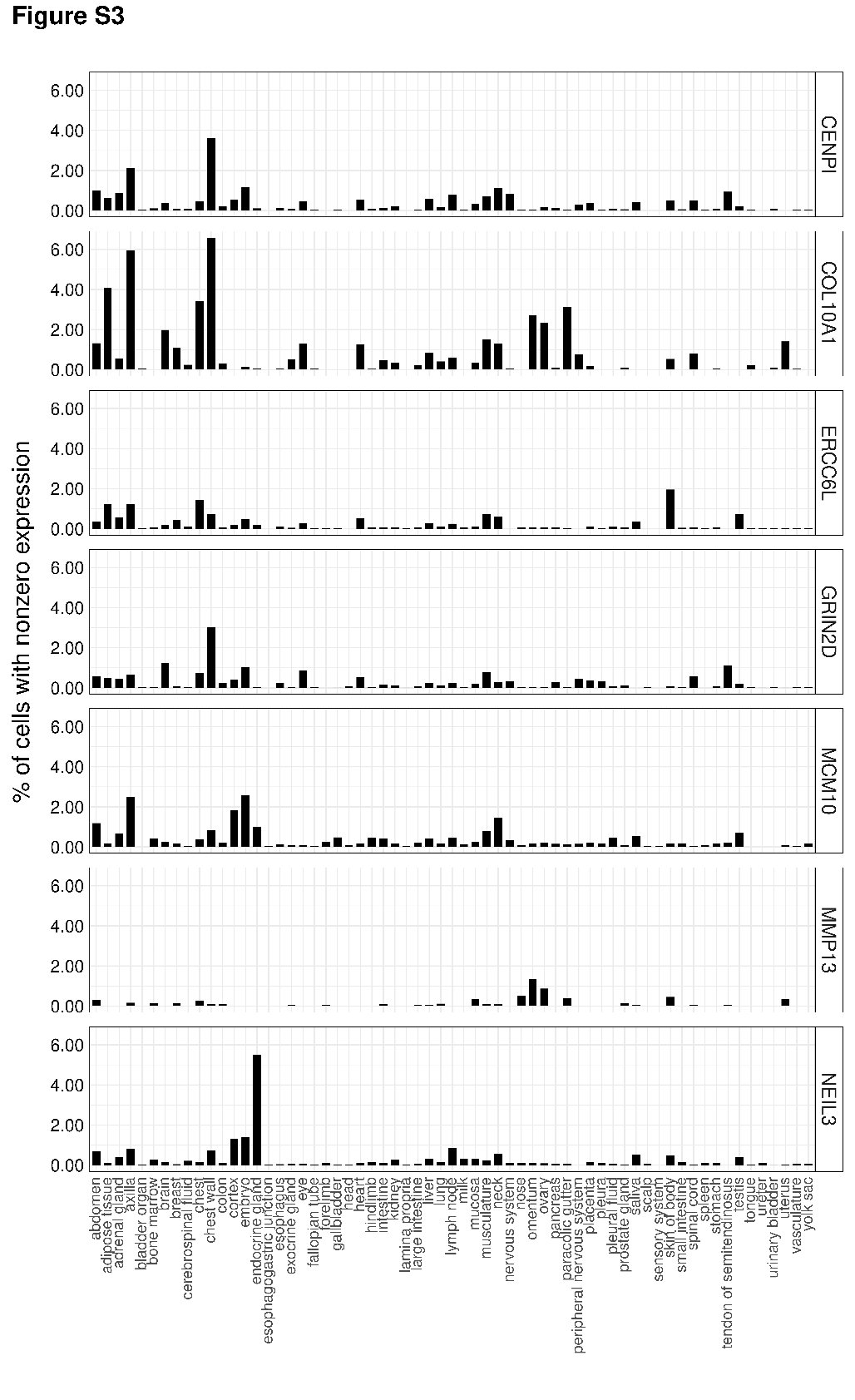


**Supplemental Figure 3**. Single-cell RNA sequencing expression proportions for 7 TAA genes across 63 non-hematopoietic sample types in the Chan Zuckerberg CELLxGENE Discover dataset, including solid tissues, anatomic sites and biological fluids. The percentage of cells with nonzero expression was calculated for each cell type as the number of cells with at least one transcript-supporting read detected for a given gene divided by the total number of cells assigned to that cell type.

**SUPPLEMENTAL METHODS**

**Selection and culture of cell lines for immunopeptidome assay**

Five cancer cell lines expressing the TAA genes described in the Results were obtained from the American Type Culture Collection (ATCC): the lung adenocarcinoma cell lines NCI-H441 (CVCL_1561), NCI-H2228 (CVCL_1543), and HCC193 (CVCL_5130); the lung squamous cell carcinoma cell line NCI-H1703 (CVCL_1490); and the breast invasive carcinoma cell line MCF-7 (CVCL_0031). All cell lines were cultured in RPMI-1640 medium (Gibco, Cat# 11875093) supplemented with 10% fetal bovine serum (Gibco, Cat #A5670701), 100 U/mL penicillin and 100 μg/mL streptomycin (Gibco, Cat# 15140122). Cell lines were passaged and maintained in the incubator at 37°C with 5% CO₂. Based on the seeding density, approximately 100 million cells were collected for each cell line. Prior to harvesting, the plates were washed three times with PBS. The cells were then scraped, collected and centrifuged at 1000 × g for 5 minutes to pellet them. After removal of the supernatant, the cell pellets were snap-frozen and stored at −80°C until further processing for the immunopeptidomic analysis.

**Sample preparation for the immunopeptidome assay**

Enrichment of HLA-bound peptides was performed as described previously (1, 2). Briefly, cell pellets were lysed in a buffer composed of PBS supplemented with 0.25% Sodium deoxycholate, 0.2 mM iodoacetamide, 1 mM EDTA, 1 mM PMSF, 1% Octyl-β-D glucopyranoside and 1:200 Protease inhibitor cocktail. The lysate was incubated on ice for 1 hour followed by centrifugation at 21,000 x g for 45 minutes at 4°C. Enrichment of HLA-peptide complexes was performed either using a KingFisher Apex automated liquid handling platform or manually. In automated mode, the lysates, Protein A-conjugated magnetic beads (Resyn Biosciences) crosslinked with pan-HLA class I specific antibody (clone W6/32), along with wash buffer A (150 mM NaCl in 20 mM Tris-HCl pH 8.0), wash buffer B (400 mM NaCl in 20 mM Tris-HCl pH 8.0) and wash buffer C (20 mM Tris-HCl pH 8.0), were transferred to 96 deep-well plates. Beads were incubated with lysate for 90 minutes with intermittent mixing speed followed by sequential washes in buffer A, buffer B, buffer A and buffer C. In manual model, Protein A-conjugated sepharose beads crosslinked with pan-HLA class I antibody were used. Finally, HLA-peptide complexes were eluted from beads using 0.5% trifluoracetic acid. Peptides were then purified using C18 spin tips, dried using a speed vac concentrator and stored at -80°C until mass spectrometry analysis.

**Liquid chromatography–tandem mass spectrometry**

For the immunopeptidome assay, peptides were reconstituted in 0.1% formic acid and analyzed either on a timsTOF Ultra 2 mass spectrometer connected online to the nanoElute 2 liquid chromatography system (Bruker Daltonik, Bremen, Germany) or on an Orbitrap Exploris 480 mass spectrometer connected to a Vanquish Neo liquid chromatography system (Thermo Scientific, San Jose, CA). The nanoElute 2 system was configured in a single column setup in which the peptides were directly loaded onto an analytical column (15 cm, 1.7 μm C_18_, IonOpticks) maintained at 45°C throughout the run. The solvent system consisted of solvent A (0.1% formic acid) and solvent B (80% acetonitrile, 0.1% formic acid) delivered at a flowrate of 300 nL/min. Peptide separation was performed using a gradient of solvent B from 2% to 28% for 85 minutes within a total run time of 95 minutes. Peptides were analyzed using DDA-PASEF mode with a polygon optimized for HLA class I peptides considering charge states 1-4. Survey MS1 scans were acquired from 100 to 1700 m/z within an ion mobility range from 1/K0 = 1.65 Vs cm^−2^ to 0.64 Vs cm^−2^. Five PASEF ramps were acquired using an accumulation and ramp time of 150 ms each. Precursor ions above the minimum intensity threshold of 500 were isolated with 2 Th at < 700 m/z or 3 Th >800 m/z for MS/MS analysis and were resequenced until reaching a target intensity of 20,000. Repeated sequencing of precursor ions was avoided using a dynamic exclusion of 0.4 minute. Isolated precursor ions were fragmented using a collision energy (CE) ramp from 55 eV at 1/K0 = 1.6 Vs cm^−2^ to 15 eV at 1/K0 = 0.6 V cm^−2^. Ion mobility and mass calibration were performed using three ESI Tuning Mix ions (m/z, 1/K0: 622.02, 0.98 Vscm^−2^, 922.01, 1.19 Vscm^−2^, 1221.99, and 1.38 Vscm^−2^).

Analysis using the Orbitrap Exploris 480 mass spectrometer was performed in data-dependent acquisition mode with a cycle time of 2 seconds. Survey MS scans were acquired in the Orbitrap mass analyzer at 60,000 resolution, with 300% normalized AGC target and 50 ms injection time. Precursor ions were isolated using quadrupole with an isolation width of 1.2 m/z and fragmented with 28% HCD normalized collision energy. Fragment ion spectra were acquired in the Orbitrap mass analyzer at 30,000 resolution, with 200% normalized AGC target and 120 ms injection time. An exclusion duration of 30 seconds was used to prevent repeated MS/MS analysis of precursors. The monoisotopic precursor selection filter was enabled along with an intensity threshold of 2.5x10^4^.

**Mass spectrometry data analysis**

The raw data were analyzed using MSFragger (version 3.5 and 4.2) from the FragPipe computational platform (versions 17.1 and 23.0, respectively). The analysis was performed in non-specific mode, allowing a peptide length of 7-25 amino acids and 20 ppm precursor and fragment ion mass tolerance against the human UniProt protein database. Oxidation at methionine and N-terminal acetylation were set as dynamic modifications. Peptide spectral matches were validated using the Percolator node with a false discovery rate of 1% at peptide level or 10% at the PSM level. The IonQuant module was enabled for quantitation of peptides identified.

**SUPPLEMENTAL REFERENCES**

1. Mangalaparthi KK, Madugundu AK, Ryan ZC, Garapati K, Peterson JA, Dey G, et al. Digging deeper into the immunopeptidome: characterization of post-translationally modified peptides presented by MHC I. J Proteins Proteom. 2021;12(3):151-60.

2. Raja R., Mangalaparthi KK, Madugundu AK, Jessen E, Pathangey L, Magtibay P, et al. Immunogenic cryptic peptides dominate the antigenic landscape of ovarian cancer. Science Advances. 2025;11(eads7405).
